# Synthesis, Biological Evaluation, and Docking Studies of a Novel Sulfonamido-Based Gallate as Pro-Chondrogenic Agent for the Treatment of Cartilage

**DOI:** 10.3390/molecules22010003

**Published:** 2016-12-23

**Authors:** Xiao Lin, Ling Chai, Buming Liu, Hailan Chen, Li Zheng, Qin Liu, Cuiwu Lin

**Affiliations:** 1 School of Chemistry and Chemical Engineering, Guangxi University, Nanning 530005, China; linxiaolegend@163.com; 2 Guangxi Key Laboratory of Traditional Chinese Medicine Quality Standards, Guangxi Institute of Traditional Medical and Pharmaceutical Sciences, Nanning 530022, China; cicichai001@163.com (L.C.); liu_buming@163.com (B.L.); hlchen319@163.com (H.C.); 3 The Medical and Scientific Research Center, Guangxi Medical University, Nanning 530021, China; zhengli224@163.com (L.Z.); liuqin_2001@163.com (Q.L.)

**Keywords:** gallic acid, sulfadiazine sodium, pro-chondrogenic agent, molecular docking

## Abstract

Gallic acid (GA) and its derivatives are anti-inflammatory agents and are reported to have potent effects on Osteoarthritis (OA) treatment. Nonetheless, it is generally accepted that the therapeutic effect and biocompatibility of GA is much weaker than its esters due to the high hydrophilicity. The therapeutic effect of GA on OA could be improved if certain structural modifications were made to increase its hydrophobicity. In this study, a novel sulfonamido-based gallate was synthesized by bonding sulfonamide with GA, and its biological evaluations on OA were investigated. Results show that 5-[4-(Pyrimidin-2-ylsulfamoylphenyl)]-carbamoyl-benzene-1,2,3-triyl triacetate (HAMDC) was able to reverse the effects induced by Interleukin-1 (IL-1) stimulation, and it also had a great effect on chondro-protection via promoting cell proliferation and maintaining the phenotype of articular chondrocytes, as well as enhancing synthesis of cartilage specific markers such as aggrecan, collagen II and Sox9. Furthermore, a docking study showed that HAMDC fits into the core of the active site of a disintegrin and metalloproteinase with thrombospondin motifs 5 (ADAMTS-5), which provides an explanation for its activity and selectivity.

## 1. Introduction

Osteoarthritis (OA), the most common form of arthritis, frequently occurring after middle age, is usually characterized by progressive failure of the extracellular cartilage matrix (ECM) and changes in the synovium and subchondral bone [1,2]. At the present time, the treatment of OA is limited to steroidal and non-steroidal anti-inflammatory drugs (NSAIDS), which merely provide symptomatic relief of pain and inflammation but cannot alleviate or slow down the progression of the disease [3]. For this reason, great efforts have been devoted to the discovery and development of new drugs to reduce the progression of OA.

In OA patients and animal models, chondrocytes usually present changes of phenotype and gene repertoire, which is fundamentally caused by abnormal excretion of biochemical factors, such as matrix metalloproteases (MMPs), aggrecanases (ADAMTS-4 and ADAMTS-5), IL-1β and other cytokines [4,5]. MMP-1 plays an important role in degrading the components of cartilage matrix, especially aggrecans and collagens. Active stromelysin (MMP-3) serves as an activator of latent collagenases and also digests proteoglycan aggregates in human articular cartilage [6]. Tissue inhibitor of matrix metalloproteinases (TIMPs) are inhibitors of MMPs and an increase of TIMP levels in OA cartilage possibly reflect an endogenous adaptive response towards the increase of proteinase activity [7]. Recently, a series of new arylsulfonamido-based hydroxamates have been synthesized and several of them exhibit a marked inhibiting effect on MMP-1, MMP-13 and MMP-14 [8]. It was further reported that some sulfonamido-based analogs show strong inhibitory activity on MMP-13 and aggrecanase [9,10]. These synthetic compounds have several phenyl and sulfonamide groups, the unique structure of which plays a leading role in the prevention of cartilage degradation. These studies shed light on the synthesis of analogs to slow down or block cartilage degradation.

Gallic acid (GA) is a well-known polyphenol and ubiquitous in tea [11], grapes [12], berries [13], and other plant species [14,15]. GA has a wide range of pharmacological activity and takes part in several pharmacological and biochemical pathways [16,17,18,19]. It has been reported that GA exhibits strong pro-apoptotic and anti-inflammatory effects in the therapy of arthritis [13,20]. The chondroprotective and chondrogenic effect of GA has also been confirmed [21,22]. Sulfonamides are a family of broad-spectrum synthetic bacteriostatic antibiotics. Due to their easy penetration across the biological membranes and transfer to targeted cells or organs, sulfonamides have been widely applied on human and animals for therapeutic and prophylactic purposes in the last century [23]. It has been reported that several sulfonamides exhibited a significant effect on the treatment of degenerative joint disorders and joint inflammation, such as osteoarthritis and rheumatism [24,25]. All these reported compounds contain several phenyl groups and sulfonamide groups, which shed light on the synthesis of derivatives by coupling GA with sulfonamides. The modification of GA with sulfonamide was expected to enhance the pharmacological activity of GA.

Recently, we have reported a series of synthesized sulfonamido-based gallates and their effectiveness on chondro-protection [26,27,28,29,30], which confirm the fact that coupling of the phenyl ring with the sulfonamide group is critical for the activity of preventing cartilage degeneration. Herein, another analog was synthesized and shown to be effective in biological evaluation, a docking study was therefore performed to provide an explanation for its activity and selectivity.

## 2. Results

### 2.1. Chemistry

**Figure 1:**
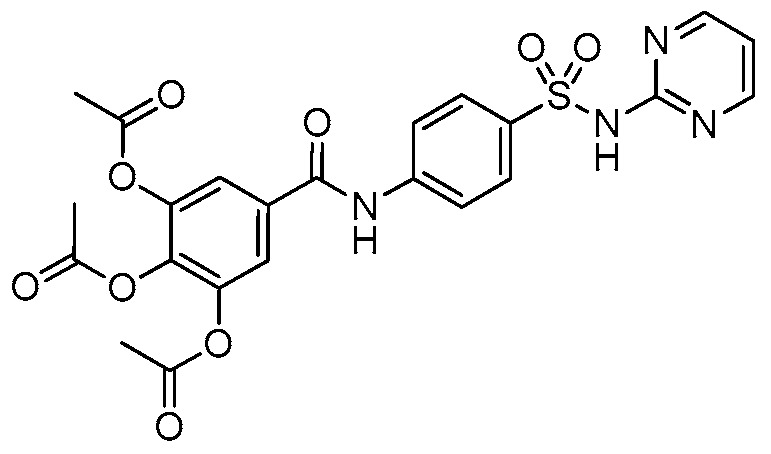
Chemical structure of HAMDC.

**Scheme 1:**
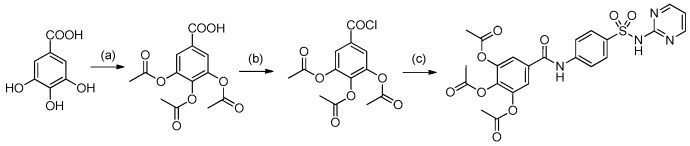
Schematic of the HAMDC synthetic procedures. *Reagents and conditions*: (**a**) acetyl oxide, oil bath, 120 °C; (**b**) SOCl_2_, oil bath, 80 °C; (**c**) sulfadiazine sodium, THF, pyridine, ice bath.

5-[4-(Pyrimidin-2-ylsulfamoylphenyl)]-carbamoyl-benzene-1,2,3-triyl triacetate (HAMDC, Figure 1) was synthesized from GA and sulfadiazine sodium following the synthetic route (Scheme 1).

The molecular formula of HAMDC was confirmed to be C_19_H_18_N_4_O_6_S by the analysis of MS-ESI (Shimadzu LC-MS 2010A) and NMR (Bruker AM-400 MHz) data (Supplementary Materials, Figures S1–S3). MS-ESI: *m*/*z*: 527.0 [M − H]^−^, ^1^H-NMR (400 MHz, DMSO) spectrum showed signals at δ: 11.75 (br s, 1H, -SO_2_-NH), 10.67 (s, 1H, -CO-NH), 8.49 (d, *J* = 4.9 Hz, 2H, Py-H), 7.95 (dd, *J* = 9.0 Hz, 4H, 4× Ar-H), 7.80 (s, 2H, 2× Ar-H), 7.03 (m, 1H, Py-H), 2.33 (s, 3H, -CO-CH_3_), 2.31 (s, 6H, -CO-CH_3_); ^13^C-NMR (100 MHz, DMSO) spectrum showed signals at δ: 168.00, 166.95, 163.66, 158.38, 156.93, 143.17, 142.72, 137.62, 134.43, 132.43, 128.72, 120.74, 119.74, 115.85, 20.32, 19.88.

### 2.2. Cytotoxicity Assay

**Figure 2:**
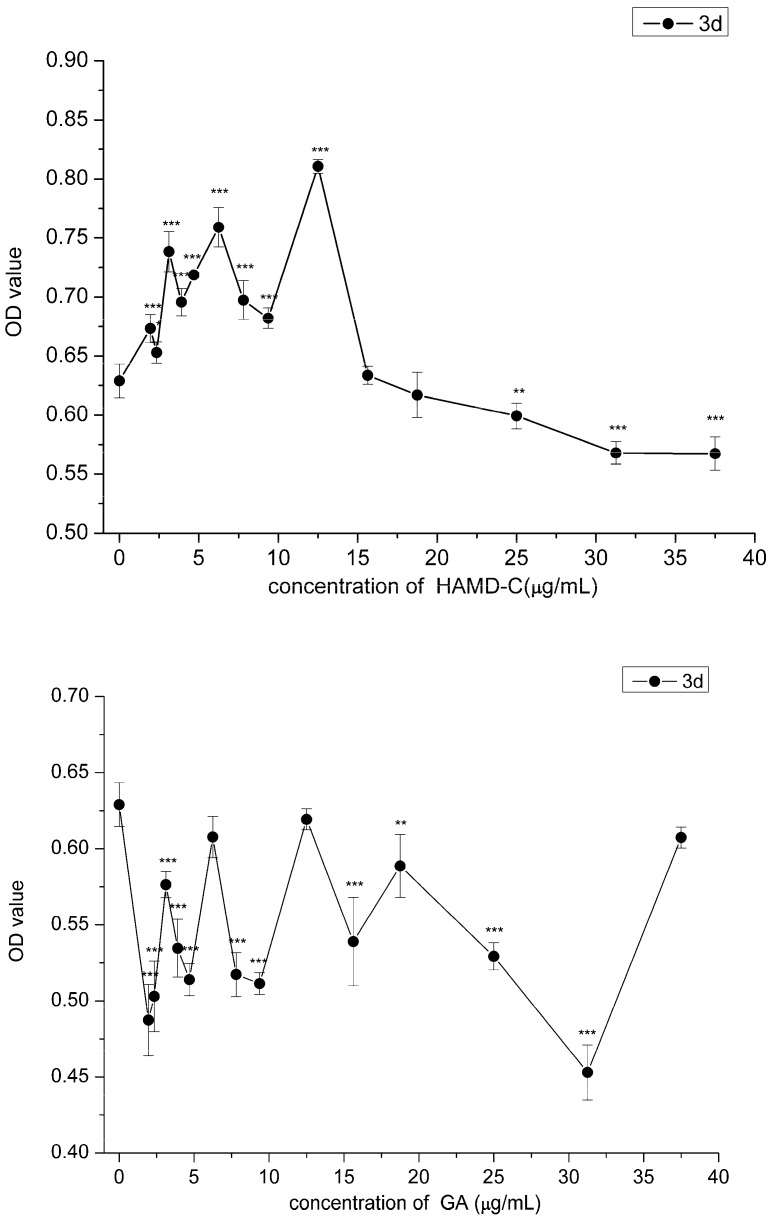

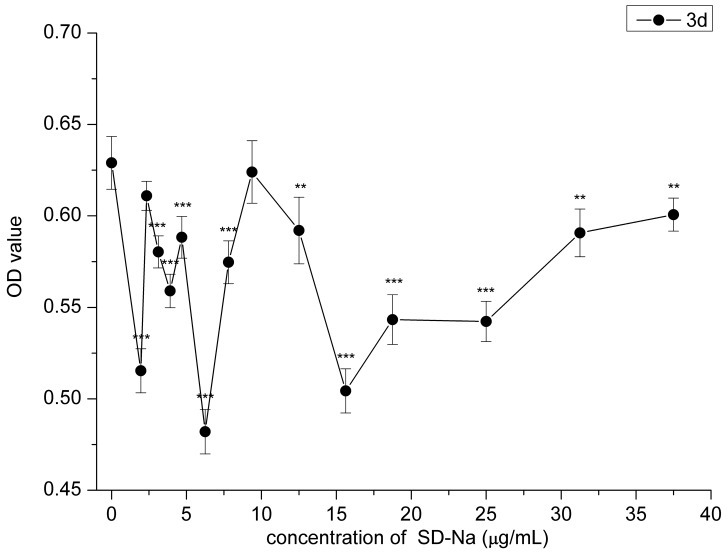
Cytotoxicity of chondrocytes treated with different concentrations of HAMDC, gallic acid (GA) and SD-Na for three days (means ± SD, *n* = 3). (* *p* < 0.05; ** *p* < 0.01, *** *p* < 0.001).

The cytotoxicity of different drugs on rabbit articular chondrocytes was investigated via MTT assays. As shown in Figure 2, negligible cycotoxicity was observed when concentrations of HAMDC between 1.950 and 15.625 μg/mL were used. However, when the concentration increased to a range between 18.750 and 37.500 μg/mL, the cell proliferation was partially inhibited. Comparatively, SD-Na and GA exhibited insignificant or certain level of inhibitive effect on chondrocytes growth depending on the drug concentrations. It is possible to conclude from this experiment that concentrations of HAMDC in the range of 3.125–12.500 μg/mL promoted cell growth, and thus concentrations of HAMDC, SD-Na, and GA were chosen as 3.125–12.500, 2.344–9.375, and 3.125–12.500 μg/mL, respectively, and were adopted in the subsequent studies.

### 2.3. Effect of HAMDC, SD-Na and GA on IL-1β Stimulated Chondrocytes

**Figure 3:**
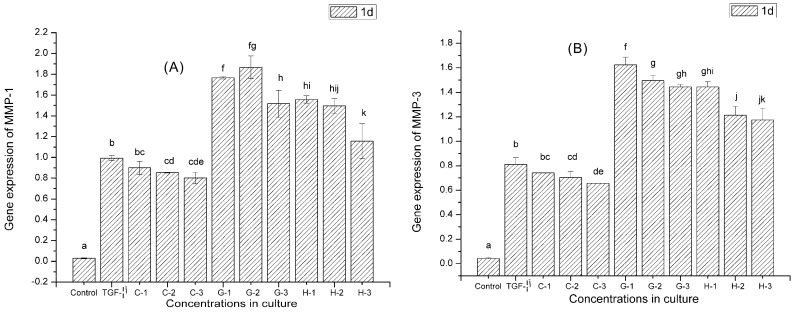

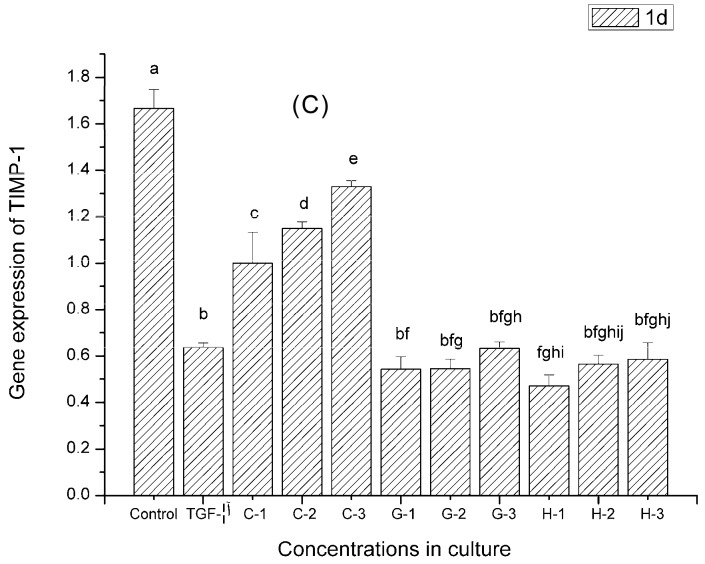
Quantitative analysis of ECM-related gene expressions by qRT-PCR, (**A**) MMP-1; (**B**) MMP-3; and (**C**) TIMP-1. Chondrocytes were cultured with different concentrations of HAMDC, GA and SD-Na for 2, 4 and 6 days. They were then stimulated with IL-1β for another 24 h. Control: cells without any treatment; IL-1β: cells stimulated with IL-1β; C-1 to C-3: cells pre-incubated with 3.125, 6.250, and 12.500 μg/mL HAMDC, respectively; G-1 to G-3: cells pre-incubated with 3.125, 6.250, and 12.500 μg/mL GA, respectively; H-1 to H-3: cells pre-incubated with 2.344, 4.688, and 9.375 μg/mL SD-Na, respectively. The gene expressions of MMP-1, MMP-3, and TIMP-1 in cells treated with HAMDC, GA and SD-Na relative to control were analyzed by the 2^−ΔΔ*C*t^ method using GAPDH as the internal control. Each condition was repeated for three times, and data are presented as mean ± SD. (*p* < 0.05).

**Figure 4:**
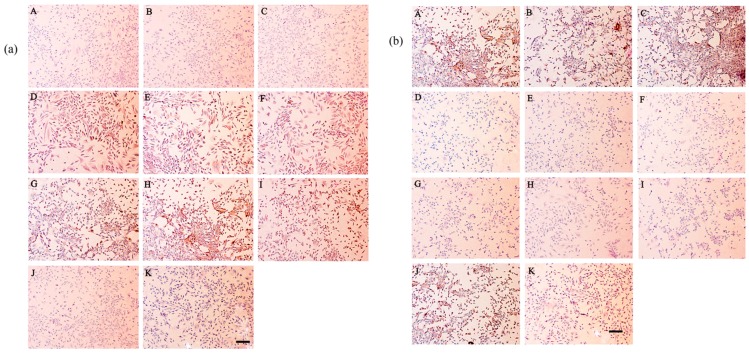
Immunohistochemical staining images of MMP-1 (**a**) and TIMP-1 (**b**). Chondrocytes were firstly pre-incubated with different concentrations of HAMDC, GA and SD-Na for 1 h, and further stimulated with IL-1β for 1 days: (**A**) 3.125 μg/mL HAMDC; (**B**) 6.250 μg/mL HAMDC; (**C**) 12.500 μg/mL HAMDC; (**D**) 3.125 μg/mL GA; (**E**) 6.250 μg/mL GA; (**F**) 12.500 μg/mL GA; (**G**) 2.344 μg/mL SD-Na; (**H**) 4.688 μg/mL SD-Na; (**I**) 9.375 μg/mL SD-Na; (**J**) Control (no drug treatment and no stimulation); (**K**) IL-1β model (no drug treatment, but with IL-1β stimulation). Scale bar = 200 μm.

To investigate their effects on arthritis, chondrocytes were pre-incubated with HAMDC, SD-Na and GA for 1 h before IL-1β stimulation. In the OA model group (with IL-1β stimulation but without drug treatment), the expressions of MMP-1 and MMP-3 were upregulated, while that of TIMP-1 was reduced (Figure 3). Pre-incubation with HAMDC inhibited the IL-1β-stimulated increase of MMP-1 and MMP-3 gene expression and significantly enhanced the TIMP-1 expression. However, pre-incubation with SD-Na and GA could not prevent the IL-1β stimulated MMP-1, MMP-3 and TIMP-1 expression. The effect of IL-1β, HAMDC, SD-Na and GA on the secretion of MMP-1 and TIMP-1 proteins from chondrocytes were tested subsequently. It was found that IL-1β stimulation resulted in upregulation of MMP-1 secretion and downregulation of TIMP-1 secretion (Figure 4). In agreement with the RT-PCR results, HAMDC was able to downregulate MMP-1 expression and upregulate TIMP-1 expression, as demonstrated with increased positive staining of MMP-1 and negative staining of TIMP-1 in the immunohistochemical images (Figure 4). These results indicated that the MMPs induction and TIMP-1 downregulation that resulted from IL-1β stimulation could be effectively blocked by HAMDC but not SD-Na and GA.

### 2.4. Cell Proliferation

**Figure 5:**
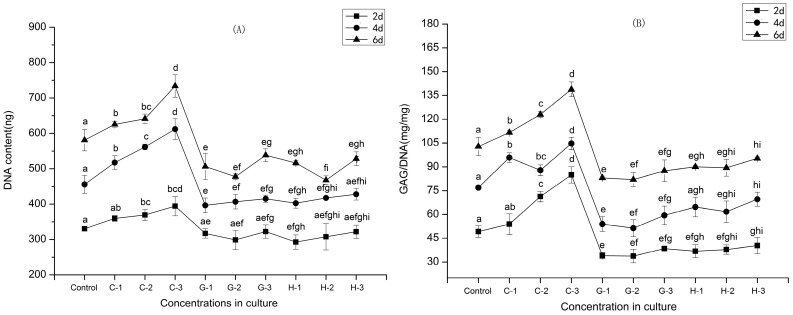
Quantification of cell proliferation (DNA) and matrix production (glycosaminoglycan (GAG)) by biochemical assays: (**A**) the proliferation of chondrocytes cultured with different concentrations of HAMDC, GA and SD-Na for 2, 4 and 6 days. Control: cells without any treatment; IL-1β: cells stimulated with IL-1β; C-1 to C-3: cells pre-incubated with 3.125, 6.250, and 12.500 μg/mL HAMDC, respectively; G-1 to G-3: cells pre-incubated with 3.125, 6.250, and 12.500 μg/mL GA, respectively; H-1 to H-3: cells pre-incubated with 2.344, 4.688, and 9.375 μg/mL SD-Na, respectively; (**B**) Content of GAG (mg) normalized to DNA (mg). Each condition was repeated for four times, and data are presented as mean ± SD. (*p* < 0.05).

In this study, the cell proliferation was analyzed by the DNA content after treatment with drugs for 2, 4, and 6 days. From Figure 5A, significantly higher DNA content has been observed from chondrocytes cultured with HAMDC comparing with control (*p* < 0.05), indicating that HAMDC was able to promote cell proliferation. The optimal concentration was found to be 12.500 μg/mL, which is in agreement with MTT results. 

### 2.5. Secretion of GAGs

Glycosaminoglycans (GAGs) are the building blocks of cartilage and joint fluid. It is thought that the secretion of glycosaminoglycans in the cartilage inhibits the enzymes that destroy cartilage. To examine the effect of HAMDC on the secretion of GAG, the chondrocytes were treated with HAMDC, SD-Na or GA for 2, 4, and 6 days and then subjected to biochemical assays. Under the same conditions, GAG production was gradually increased over time (Figure 5B). It was found that HAMDC treatment dramatically enhanced the GAG production compared to controls. In contrast, SD-Na and GA treatment reduced the GAG production. In line with the cell proliferation results, a concentration of HAMDC at 12.5 μg/mL exhibited the strongest effect on promoting GAG synthesis among the three tested concentrations.

**Figure 6:**
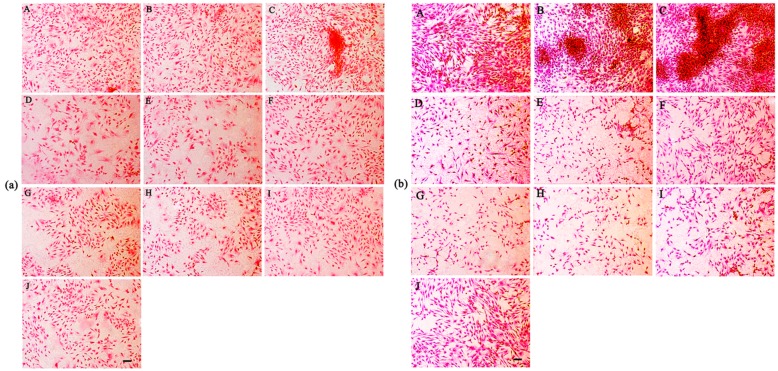
Inverted phase contrast microscopy images showing the GAG production (**a**) and cellular morphology (**b**) of chondrocytes treated with different concentrations of HAMDC, GA and SD-Na for 6 days: (**A**) 3.125 μg/mL HAMDC; (**B**) 6.250 μg/mL HAMDC; (**C**) 12.500 μg/mL HAMDC; (**D**) 3.125 μg/mL GA; (**E**) 6.250 μg/mL GA; (**F**) 12.500 μg/mL GA; (**G**) 2.344 μg/mL SD-Na; (**H**) 4.688 μg/mL SD-Na; (**I**) 9.375 μg/mL SD-Na; (**J**) Control (no drug treatment and no stimulation). Scale bar = 200 μm.

The safranin *O*-positive stain (Figure 6a) shows that GAGs were abundant and homogeneously distributed around the chondrocytes which have been treated with HAMDC. In contrast, fewer GAGs have been observed in condrocytes treated with SD-Na and GA compared to the control.

### 2.6. Cell Morphology

The morphology of articular chondrocytes treated with HAMDC (3.125, 6.250 and 12.500 μg/mL), SD-Na (2.344, 4.688 and 9.375 μg/mL) and GA (3.125, 6.250 and 12.500 μg/mL) for 2, 4, and 6 days were observed under an invert microscope (Figure 6b, here shows cells treated for 6 days). The number of condrocytes in the HAMDC group was obviously larger than the SD-Na, GA, and control groups. Cells with all tested concentrations of HAMDC-grown confluent were observed for 2 days with healthy morphology (data not shown), and the largest number of cells was obtained when a concentration of 12.500 μg/mL was used. In contrast, the above phenomenon was not observed in SD-Na and GA groups.

**Figure 7:**
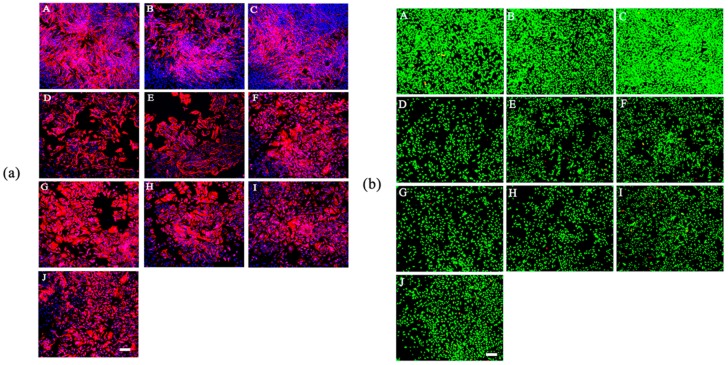
Confocal laser scanning microscopy images showing the actin filaments (**a**) and viability (**b**) of chondrocytes treated with different concentrations of HAMDC, GA and SD-Na for 6 days: (**A**) 3.125 μg/mL HAMDC; (**B**) 6.250 μg/mL HAMDC; (**C**) 12.500 μg/mL HAMDC; (**D**) 3.125 μg/mL GA; (**E**) 6.250 μg/mL GA; (**F**) 12.500 μg/mL GA; (**G**) 2.344 μg/mL SD-Na; (**H**) 4.688 μg/mL SD-Na; (**I**) 9.375 μg/mL SD-Na; (**J**) Control (no drug treatment and no stimulation). Scale bar = 200 μm.

Figure 7a showed the actin filaments of chondrocytes stained with rhodamine phalloidin/Hoechst 33258. A higher density and larger covered area of filaments was observed from the HAMDC groups compared with other conditions, which was in agreement with the HE analysis (Figure 6b).

### 2.7. Cell Viability Assay

The viable and dead cells were determined by the calcein/PI staining method (Figure 7b). It seems that the number of live cells in the HAMDC groups was much larger than that of the SD-Na, GA group, and control groups, which consistent with the cell proliferation results. These findings suggest that HAMDC might benefit from the chondrocyte survival and growth, and the optimal concentration is 12.500 μg/mL.

### 2.8. Secretion of Type I and Type II Collagen

**Figure 8:**
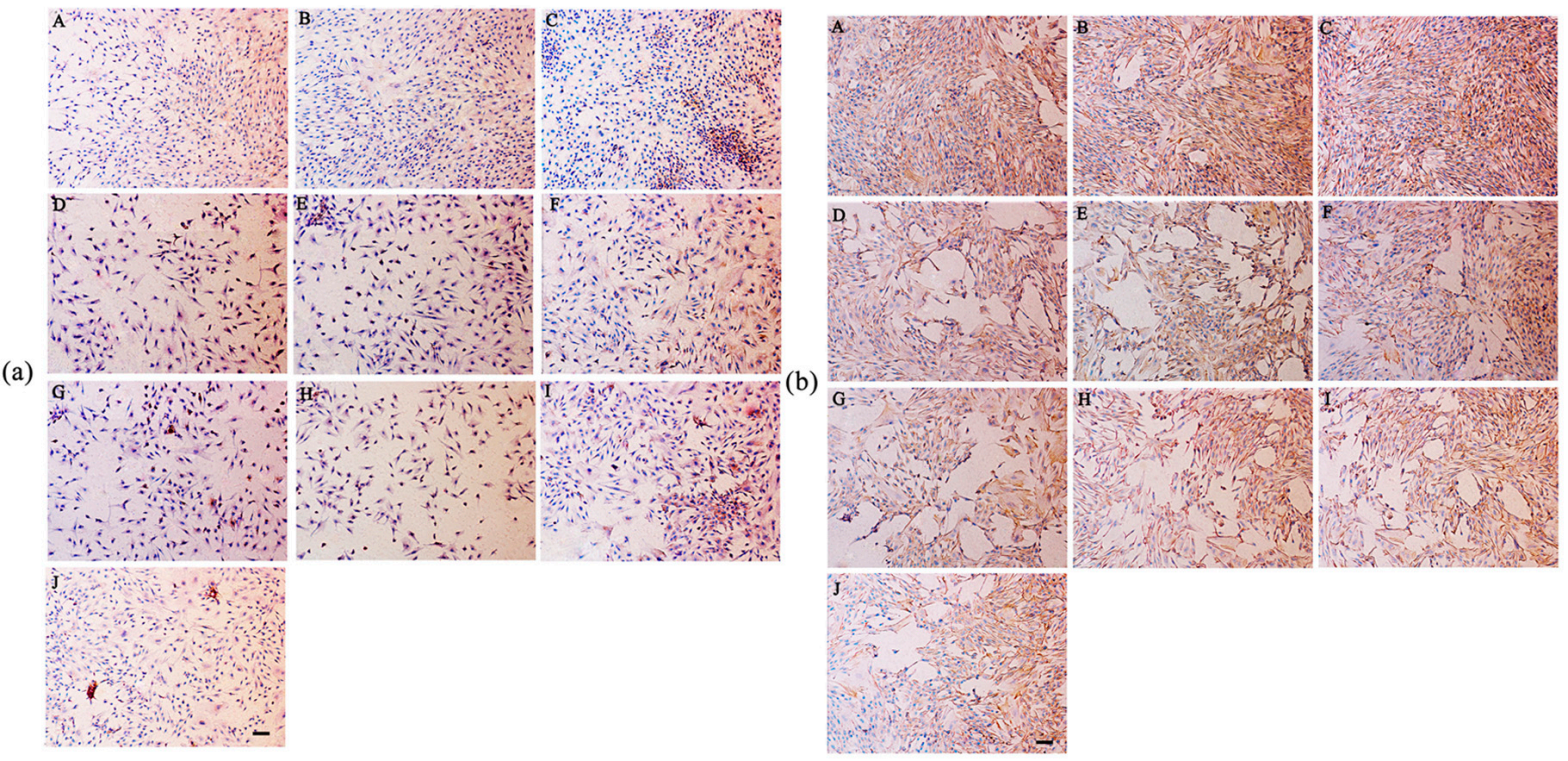
Immunohistochemical staining images showing the presence of type I (**a**) and type II (**b**) collagens. Chondrocytes treated with different concentrations of HAMDC, GA and SD-Na for 6 days: (**A**) 3.125 μg/mL HAMDC; (**B**) 6.250 μg/mL HAMDC; (**C**) 12.500 μg/mL HAMDC; (**D**) 3.125 μg/mL GA; (**E**) 6.250 μg/mL GA; (**F**) 12.500 μg/mL GA; (**G**) 2.344 μg/mL SD-Na; (**H**) 4.688 μg/mL SD-Na; (**I**) 9.375 μg/mL SD-Na; (**J**) Control (no drug treatment and no stimulation). Scale bar = 200 μm.

The cartilaginous intracellular matrix deposition was evaluated by immunohistochemical assay of type I and type II collagen. From Figure 8, a large area of positive stained cartilage-specific type II collagen was observed in the HAMDC groups after 2, 4, and 6 days. In contrast, sparse and light stained type I collagen was observed, indicating the inhibition of chondroncyte dedifferentiation. For SD-Na and GA groups, type II collagen staining was weaker and collagen I staining was stronger compared to the control. These results imply that HAMDC possesses greater ability to inhibit chondrocyte dedifferentiation in vitro compared to SD-Na and GA.

### 2.9. Gene Expression

**Figure 9:**
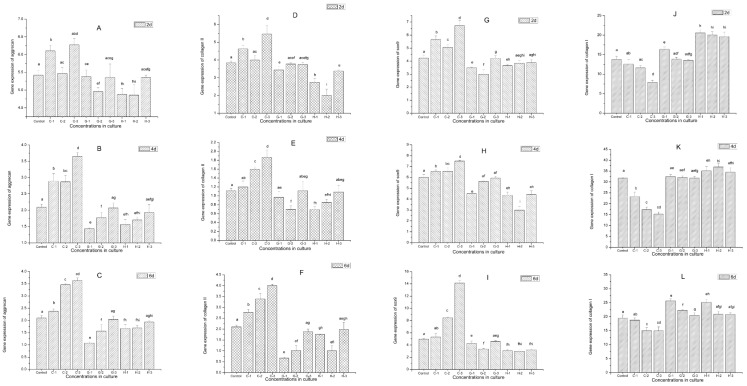
Quantitative comparison of ECM-related gene expression by qRT-PCR: (**A**–**C**) aggrecan; (**D**–**F**) collagen II; (**G**–**I**) Sox9; and (**J**–**L**) collagen I. The chondrocytes were pre-incubated with different concentrations of HAMDC, GA and SD-Na for 2 days, 4 days and 6 days, and then stimulated by 10 ng/mL IL-1β. Control: cells without any treatment; IL-1β: cells stimulated with IL-1β; C-1 to C-3: cells pre-incubated with 3.125, 6.250, and 12.500 μg/mL HAMDC, respectively; G-1 to G-3: cells pre-incubated with 3.125, 6.250, and 12.500 μg/mL GA, respectively; H-1 to H-3: cells pre-incubated with 2.344, 4.688, and 9.375 μg/mL SD-Na, respectively. The gene expressions of aggrecan, collegen II, Sox9 and collagen I in cells treated with HAMDC, GA and SD-Na relative to control were analyzed by the 2^−ΔΔ*C*t^ method using GAPDH as the internal control. Each condition was repeated for three times, and data are presented as mean ± SD (*p* < 0.05).

The effect of HAMDC, SD-Na and GA on chondrocyte ECM synthesis was further investigated by detecting the gene expression of collagen I, collagen II, collagen X, Sox9, and aggrecan (a proteoglycan composed of GAGs). Figure 9 showed that the transcription of aggrecan, collagen II, and Sox9 were notably promoted by HAMDC but significantly suppressed by SD-Na and GA. Compared to the control, lower collagen I expression was observed from the HAMDC group but higher or similar levels of collagen I expression were found from the SD-Na and GA groups. Particularly, cells treated with 12.500 μg/mL of HAMDC showed the best performance with highest levels of collagen II, aggrecan, and Sox9 expression. Meanwhile, collagen X could hardly be detected under all conditions, suggesting the absence of cell hypertrophy. These results indicate that HAMDC was able to upregulate the expression of collagen II, aggrecan, and Sox9 and downregulate that of collagen type I, indicating that HAMDC might be able to delay or prevent the chondrocyte dedifferentiation.

### 2.10. Molecular Docking Studies of Sulfonamido-Based Gallates

**Figure 10:**
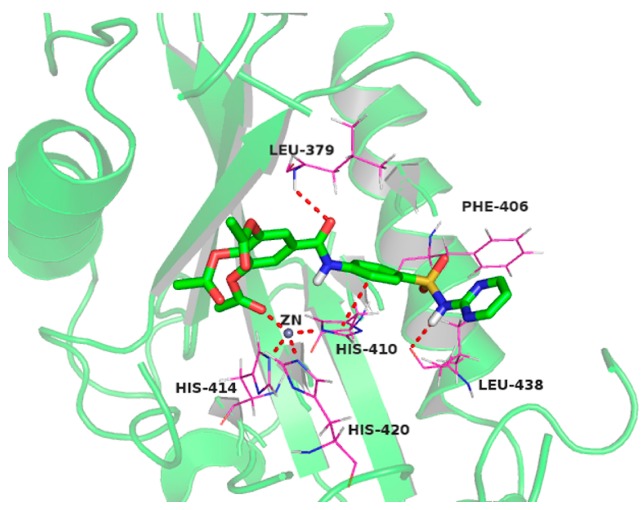
Docked pose of HAMDC with ADAMTS-5 (PDB code: 3B8Z).

**Figure 11:**
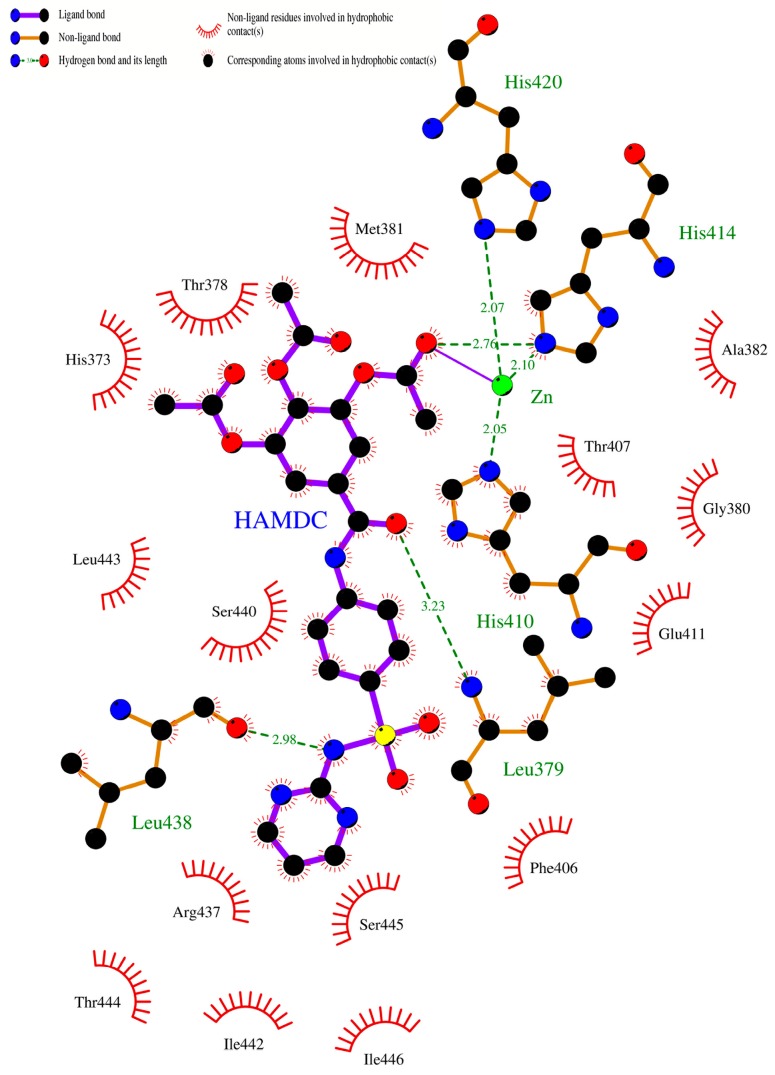
The interaction between HAMDC and ADAMTS-5 (PDB code: 3B8Z) on a 2D Diagram.

It is envisaged that the sulfonamido-based gallates that have been demonstrated to be effective might owe this to their binding interaction with the ADAMTS-5 L-shaped S1’ subsite. In light of this hypothesis, we performed molecular docking to find out whether this proposal is reliable. The catalytic site in ADAMTS-5 structure has an L-shaped funnel, opening up at the zinc site at its widest point, the hydrophobic channel through the S1’-site and out to the solvent at its distalend. In our docking result (Figure 10), HAMDC is in the lowest energy docked conformation, the acetyl group of gallic acid moiety coordinates the catalytic zinc atom in a chelation fashion with H410, H414, and H420, and establishes three hydrogen bonds with the H414, L379, and L438 side chain. Meanwhile, the benzyl ring of benzsulfamide establishes a pi-pi interaction with the imidazole ring of H410. Furthermore, other lipophilic contacts are detectable between HAMDC and the side chain of H373, T378, S440, S445, and R437 as seen in Figure 11. Clearly, all these interactions endowed HAMDC with a inhibitory activity toward ADAMTS-5.

## 3. Discussion

In Nuti’s report [8], the phenyl ring adjacent to the sulfonamide group is important for the pharmacological activity of arylsulfonamide hydroxamate, since it allows the perfect docking of sulfonamides onto ADAMTS-5 and then inhibits ADAMTS-5 activity on the native aggrecan substrate. In this study, a novel compound (HAMDC) was synthesized from GA and sulfonamide. Its effect on IL-1β stimulated rabbit articular chondrocytes has been studied. It was found that HAMDC effectively inhibited the IL-1β-stimulated induction of MMP-1 and MMP-3 but enhanced the expression of TIMP-1. However, a negligible effect has been observed when GA and SD-Na were used. These results confirm the fact that coupling of phenyl ring with the sulfonamide group is critical for the activity of the compound and the GA modification is helpful to improve its pharmacological activity. It can be concluded form this study that HAMDC has great potential to be developed as a potent anti-inflammatory agent for the OA treatment.

On the other hand, our research indicates that HAMDC was able to promote chondrocyte growth, showing better results compared to the control and its raw materials (Figure 2). Biochemical assays have shown that HAMDC could greatly promote GAG deposition around cultured chondrocytes (Figure 5). Further, HAMDC possesses the ability to upregulate the expression of aggrecan, collagen II, and Sox9 (Figure 9). All these results suggest that HAMDC can benefit chondrocyte proliferation and stimulate exuberant cartilage matrix secretion. In contrast, the expression of collagen type I, a marker of chondrocyte dedifferentiation, was effectively inhibited by HAMDC. Moreover, collagen type X, a protein specifically reflecting hypertrophic chondrocytes and regulating the onset of endochondral ossification, was nearly undetectable in the HAMDC groups. Therefore, the reduced transcription of collagen I and undetectable collagen X demonstrate that HAMDC might be able to prevent the chondrocyte dedifferentiation and hypertrophy.

Meanwhile, Molecular docking was conducted. With its acetyl moiety coordinating the catalytic zinc atom, three established hydrogen bonds, and a pi-pi interaction with the imidazole ring, the HAMDC presented a good docking result with the ADAMTS-5 L-shaped S1’ subsite. The deacetylation derivative of HAMDC was also acquired in an acid hydrolysis experiment after HAMDC was purified and scored a high affinity in the docking study. However, it exhibited an inhibitive effect on chondrocytes growth in the concentration between 0 and 40 μg/mL in a cytotoxicity assay, and therefore the subsequent experiments were not performed in this way. Nevertheless, other synthesized sulfonamido-based gallate analogues we had investigated previously [26,27,28,29,30] presented great effect on promoting chondrocyte growth and upregulating the expression of aggrecan, collagen II and Sox9, which confirm the fact that coupling of the phenyl ring with the sulfonamide group is critical for the pro-chondrogenic activity of these compounds and this docking model would provide us with a quick method to screen out the effective compounds. More docking studies should be performed in our further investigation to optimize their structures.

## 4. Materials and Methods 

### 4.1. Synthesis and Preparation of HAMDC

HAMDC was synthesized from GA and sulfadiazine sodium following the synthetic route which was reported previously [27]. Five grams of GA was dissolved in 20 mL acetyl oxide and refluxed for 8 h. Subsequently, 300 mL distilled water was added to produce recipitates. The precipitates were filtrated and dried, then 20 mL SOCl_2_ was added and refluxed for 6 h. The solution was evaporated in vacuo and mixed with 8 g sulfadiazine sodium in THF and stirred in an ice bath for 2 h. Upon the end of the reactions, an appropriate volume of distilled water (~10 times to the volume of reaction system) was added to the mixture. The obtained product was precipitated and filtered. It was then recrystallized in a THF-methanol solvent system and dried in a vacuum oven at 80 °C. The obtained product exhibited a pale yellow color in a powder form. The yield of HAMDC was 61% (3.05 g).

To prepare the stock solution (20 mg/mL), 20 mg HAMDC was dissolved in 0.1 M sodium hydroxide solution (NaOH, Sigma, Saint Louis, MO, USA), then the pH was adjusted to 7.0 with 0.1M HCl, and finally diluted with water to 1 mL. Aqueous stock solutions of GA (20 mg/mL) and sulfadiazine sodium (SD-Na) (20 mg/mL) were also prepared. All stock solutions were stored at −4 °C before being used.

### 4.2. Articular Chondrocyte Culture

The articular chondrocytes were harvested from knee joint cartilage slices of one-week-old New Zealand rabbits by enzymatic digestion. The cartilage slices were first digested with 0.25% trypsin (Solarbio, Beijing, China) for 30 min. They were then further digested with 2 mg/mL collagenase type II (Gibco, Big Cabin, OK, USA) in alpha-modified Eagle’s medium (α-MEM, Gibco) for 3 h. Cells were collected via centrifugation in 1000 rpm for 5 min, followed by resuspended in a complete culture medium consisting of α-MEM supplemented with 20% (*v*/*v*) fetal bovine serum (FBS) (Gibco) and 1% (*v*/*v*) antibiotics (100 U/mL penicillin and 100 U/mL streptomycin, Gibco). Cells were maintained at 37 °C in a saturated-humid atmosphere of 95% air and 5% CO_2_. The culture medium was replaced every other day. The second passage of articular chondrocytes was used for the following experiments.

### 4.3. Cytotoxicity Assay

The cytotoxicity of HAMDC on chondrocyte was evaluated by the 3-(4,5-dimethylthiahiazol-2-y1)-2,5-di-phenyltetrazolium bromide (MTT, Gibco) assay. Specifically, articular chondrocytes were cultured in 96-well plates with 200 μL culture medium in each well. Cells were treated with HAMDC, GA and SD-Na with defined concentrations. After 3 days cultured, 20 μL of MTT stock solution (5 mg/mL, Gibco) was added to each well and further incubated for 4 h. The supernatant was carefully removed, the formazan crystals was dissolved in 150 μL of dimethyl sulfoxide (DMSO, Gibco), and the absorbance at 570 nm was measured using an enzyme-labeled meter (Thermo Fisher Scientific, Hemel Hempstead, UK). The cytotoxicity results showed that HAMDC concentrations ranging from 1.950 to 15.625 μg/mL did not have a negative effect on cell survival and proliferation. Thus, final concentrations of HAMDC and GA ranging from 3.125 to 12.500 μg/mL, SD-Na from 2.344 to 9.375 μg/mL were chosen for further analysis.

### 4.4. Effect of HAMDC on IL-1 Stimulated Chondrocytes

To investigate the effect of HAMDC, SD-Na and GA on Interleukin-1 (IL-1) stimulated chondrocytes, five groups were divided as following: (1) control group, chondrocytes without any treatment; (2) OA model group, chondrocytes treated with IL-1β (10 ng/mL, Gibco, USA); (3) HAMDC treatment groups, chondrocytes pre-incubated with three concentrations of HAMDC (3.125, 6.250 and 12.500 μg/mL) followed by stimulation with IL-1β for 24 h; (4) SD-Na treatment groups, chondrocytes pre-incubated with three concentrations of SD-Na (2.344, 4.688 and 9.375 μg/mL) followed by stimulation with IL-1β for 24 h; (5) GA treatment groups, chondrocytes pre-incubated with three concentrations of GA (3.125, 6.250 and 12.500 μg/mL) followed by stimulation with IL-1β for 24 h. The concentrations of HAMDC, SD-Na and GA were determined by cytotoxicity assay results.

### 4.5. Cell Proliferation Analysis and Biochemical Assay

Cell proliferation and biochemical assays were carried out after treating with HAMDC, GA, and SD-Na for 2, 4, and 6 days. For the cell proliferation assay, cells were first digested with proteinase K (Sigma), and their DNAs were labeled with a fluorescent dye Hoechst 33258. The fluorescence intensity at 460 nm indicating the total DNA content was measured. The total glycosaminoglycans (GAGs) under each condition were measured by the 1,9-dimethylmethylene blue (DMMB; Sigma) spectrophotometric assay following the manufacture’s instruction. Chondroitin sulfate (Sigma) was used as control to plot the standard curve. The amount of GAGs in each group was normalized to the total DNA content.

### 4.6. Safranin O Staining

The secretion of glycosaminoglycan (GAG) was confirmed by Safranin-O/fast green-stain. Specifically, cells were fixed with 95% alcohol for 30 min and then stained with 0.1% Safranin O (Sigma) for 10 min. Subsequently, the cells were rinsed with water and sealed with neutral gum. They were then observed under an inverted phase contrast microscope and photographed with adapted camera (Zeiss Corporation, Oberkochen, Germany).

### 4.7. Morphological Examination

The cellular morphology of articular chondrocytes was observed with optical microscope and fluorescent microscope. For optical microscope, cells were cultured for 2, 4, and 6 days, followed by fixing in 95% alcohol. Cells were then subjected to Hematoxylin-eosin (HE) (Jiancheng Biotech, China) staining, followed by washing with PBS, naturally dried, and sealed with neutral gum. After that, cells were observed under an inverted phase contrast microscope and photographed with an adapted camera.

Fluorescent microscope was employed to investigate the structure of cellular matrix. Firstly, cells were fixed with 4% paraformaldehyde (PFA, Sigma) for 10 min at room temperature, rinsed with PBS, and then treated with 0.5% Triton X-100 (Sigma Aldrich) for 5 min. Cells were then treated with rhodamine phalloidin (Invitrogen, Carlsbad, CA, USA) for 30 min at room temperature in dark followed by double-staining with Hoechst 33258 (Beyotime, Santa Cruz, CA, USA) for 5 min. Finally, the labeled-articular chondrocytes were observed with a laser scanning confocal microscope (Nikon A1, Tokyo, Japan).

### 4.8. Cell Viability Assay

The cell viability of articular chondrocytes cultured for 2, 4, and 6 days were studied using a live-dead viability assay kit (Invitrogen). Briefly, calcein-AM and propidium iodide were added to the cell cultures to a final concentration of 1 μmol/L for each. Cells were then incubated in dark at 37 °C for 5 min. Stained cells were observed and imaged with a laser scanning confocal microscope (Nikon A1, Japan). Cell viability were measured and calculated from the fluorescent intensity of obtained images.

### 4.9. Immunohistochemical Staining

The secretion of collagen type I and type II, MMP-1 and TIMP-1 were measured using immunohistochemical staining kits (Bioss, Beijing, China) following the manufacture’s instructions. Specifically, cells were firstly fixed in 4% (*w*/*v*) paraformaldehyde for 5 min and then treated with Triton X-100 for 5 min. To exclude endogenous peroxidase activity, cells were incubated with 3% H_2_O_2_ for 10 min and then blocked with goat serum for 10 min at room temperature. Cells were then incubated with primary collagen type I and type II (rabbit anti-rabbit antibody, Boster, Wuhan, China) for 2.5 h. They were then rinsed with PBS 3 times and incubated with second antibody attached with biotin-labeled horseradish peroxidase for 30 min. Subsequently, the cells were counterstained with hematoxylin and then analyzed using the 3′-diaminobenzidine tetrahydrochloride (DAB) kit (Boster) according to the manufacturer’s instructions. The cells were then gradually dehydrated by series concentration of ethanol and sealed with neutral gum. An inverted phase contrast microscope was used to evaluate and photograph the stained cells.

### 4.10. RNA Extraction, Real-Time Fluorescence Quantitative Reverse Transcription–Polymerase Chain Reaction (qRT-PCR)

qRT-PCR was used to analyze the expression of type I, II and X collagen, aggrecan, Sox9, MMP-1, MMP-3 and TIMP-1. The sequences of the specific primers were designed according to specific genes and shown in Table 1. Total intracellular RNA was extracted using RNA isolation kits (Tiangen Biotechnology; Beijing, China). Approximately 300 ng of total RNA was used as templates for the reverse transcription into cDNA using reverse transcription kit (Fermentas Company, Waltham, MA, USA). The qRT-PCR reactions were performed on a Quantitative PCR Detection System (Realplex 4, Eppendorf Corporation, Hauppauge, NY, USA) with a FastStart Universal SYBR Green Master (Mix, Roche Company, Grenzach, Germany) under the cycle conditions of 10 min at 95, 15 s at 95 °C and 1 min at 60 °C. The melting curve data were recorded to verify the PCR specificity. Each gene was analyzed in triplicate to diminish operation errors. The relative gene expression levels were calculated using the 2^−ΔΔ*C*t^ method according to glyceraldehyde-3-phosphate dehydrogenase (GAPDH).

### 4.11. Docking Setup

Molecular docking was performed with the aim of revealing the interaction between HAMDC and ADAMTS-5 at an atomic level. The docking of HAMDC into ADAMTS-5 (PDB ID: 3B8Z) was carried out by autodock program, The AutoDockTools 1.5.6 package was employed to generate the docking input files, PyMOL 1.6 as the graphical user interface for 3D structure visualization, and Ligplus as the 2D structure-protein interaction visualizer to show the hydrogen bonds and hydrophobic contacts. The docking centre has been defined as center_x: 12.695, center_y: 2.665, and center_z: −5.569. Grids points of 56 20 26 with 0.375 Å spacing were calculated around the docking area for all the ligand atom types using AutoGrid4. 100 separate docking calculations were performed using the Lamarckian genetic algorithm local search (GALS) method. The docking results from each of the 100 calculations were clustered on the basis of root-mean-square deviation (rmsd = 2.0 Å) between the Cartesian coordinates of the ligand atoms and were ranked on the basis of the free energy of binding. In terms of the above parameters, a re-docking process was performed as well and it presented an excellent similarity to the ligand in 3B8Z Crystal Structure, revealing that the method we developed was reliable.

## 5. Conclusions 

In conclusion, a new chemical compound was successfully synthesized by bonding sulfonamide to the GA chemical structure in this study. It was demonstrated that HAMDC was able to relieve the IL-1 stimulated chondrocyte destruction and thus lower the progression of OA. Further, HAMDC possessed the ability to promote cell proliferation and maintain the phenotype of rabbit articular chondrocytes. Summarizing the obtained results in our study, we believe that HAMDC has great potential as a pro-chondrogenic agent for the treatment of cartilage problems.

## Figures and Tables

**Table 1:** Sequences of the primers used in the qRT-PCR.

mRNA	Forward Primer	Reverse Primer
GAPDH	5′-GTCATCATCTCAGCCCCCTC-3′	5′-GGATGCGTTGCTGACAATCT-3′
Aggrecan	5′-TTGCCTTTGTGGACACCAGT-3′	5′-GAGCCAAGGACGTAAACCCA-3′
type I collagen	5′-CCCAGCCACCTCAAGAGAAG-3′	5′-CGGGGCTCTTGATGTTCTCA-3′
type II collagen	5′-TCCGGAAACCAGGACCAAAG-3′	CTTTGTCACCACGGTCACCT-3′
type X collagen	5′-CTACGCTGAGCGGTACCAAA-3′	5′-GGCTTCCCAGTGGCTGATAG-3′
Sox9	5′-GACGCACATCTCGCCCAAC-3′	5′-TCTCGCTTCAGGTCAGCCTT-3′

The results are presented as means ± SD. The statistical significance was determined using one way analysis of variance (ANOVA) followed by Dunnett’s post hoc test. The level of significance was set to *p* < 0.05.

## Supplementary Materials

Supplementary materials can be accessed at: http://www.mdpi.com/1420-3049/22/1/3/s1.
