# Supplementary material for: Synthesis, Biological Evaluation, and Docking Studies of a Novel Sulfonamido-Based Gallate as Pro-Chondrogenic Agent for the Treatment of Cartilage
Source: Molecules. 2016 Dec 23;22(1):3. doi: 10.3390/molecules22010003 (PMC6155774; doi:10.3390/molecules22010003)
Supplement: Supplementary file 1 [file molecules-22-00003-s001.pdf]

Supplementary Materials: Synthesis, Biological Evaluation, and Docking Studies of a Novel Sulfonamido-Based Gallate as Pro-Chondrogenic Agent for the Treatment of Cartilage

Xiao Lin, Ling Chai, Buming Liu, Hailan Chen, Li Zheng, Qin Liu and Cuiwu Lin

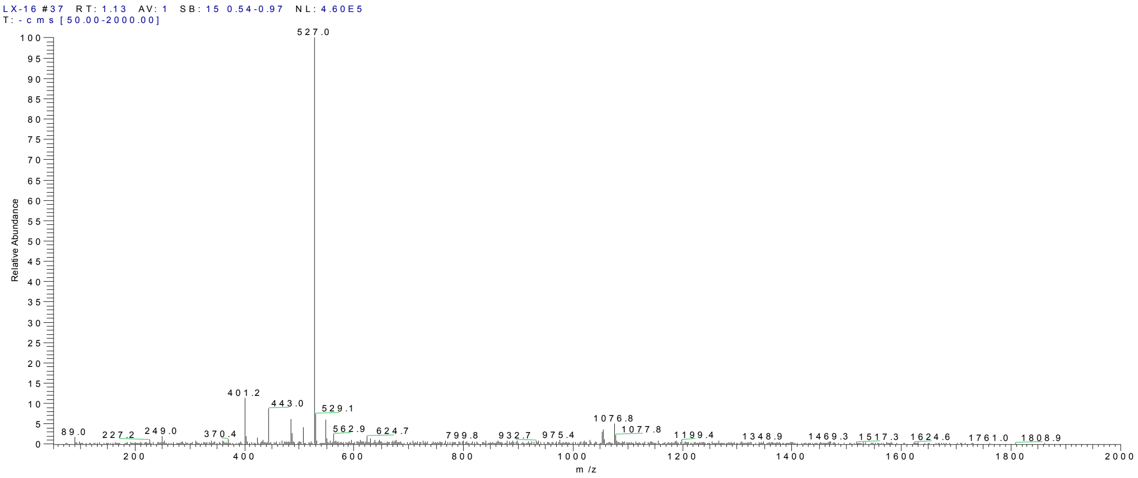

Figure S1. MS spectrum of HAMDC.

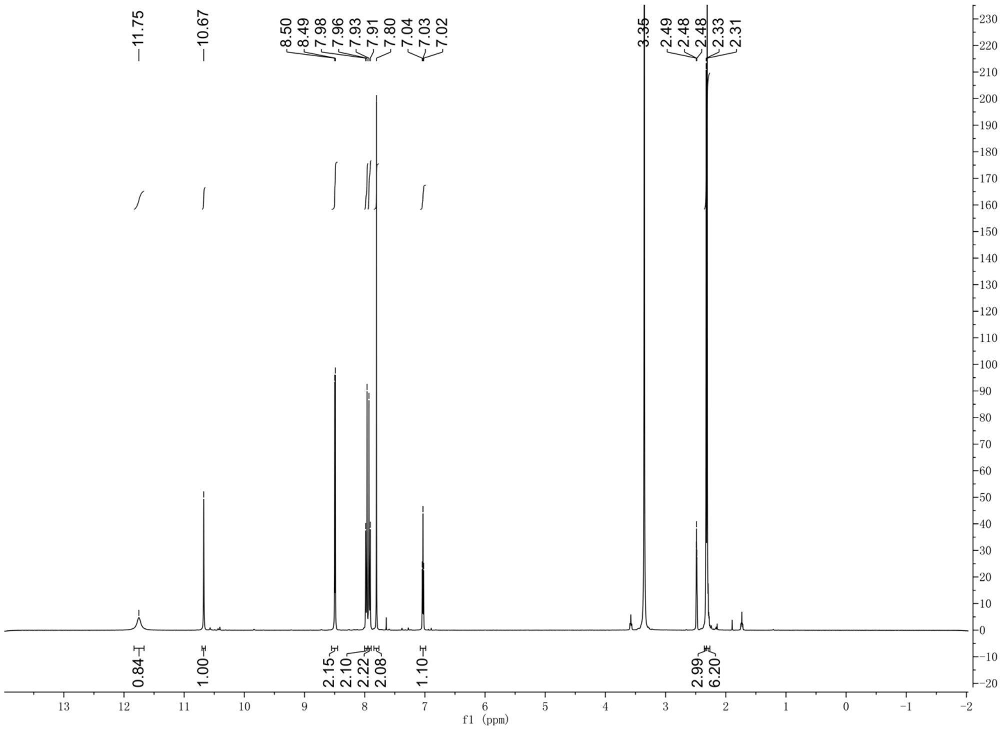

Figure S2. ^{1}H-NMR spectrum of HAMDC.

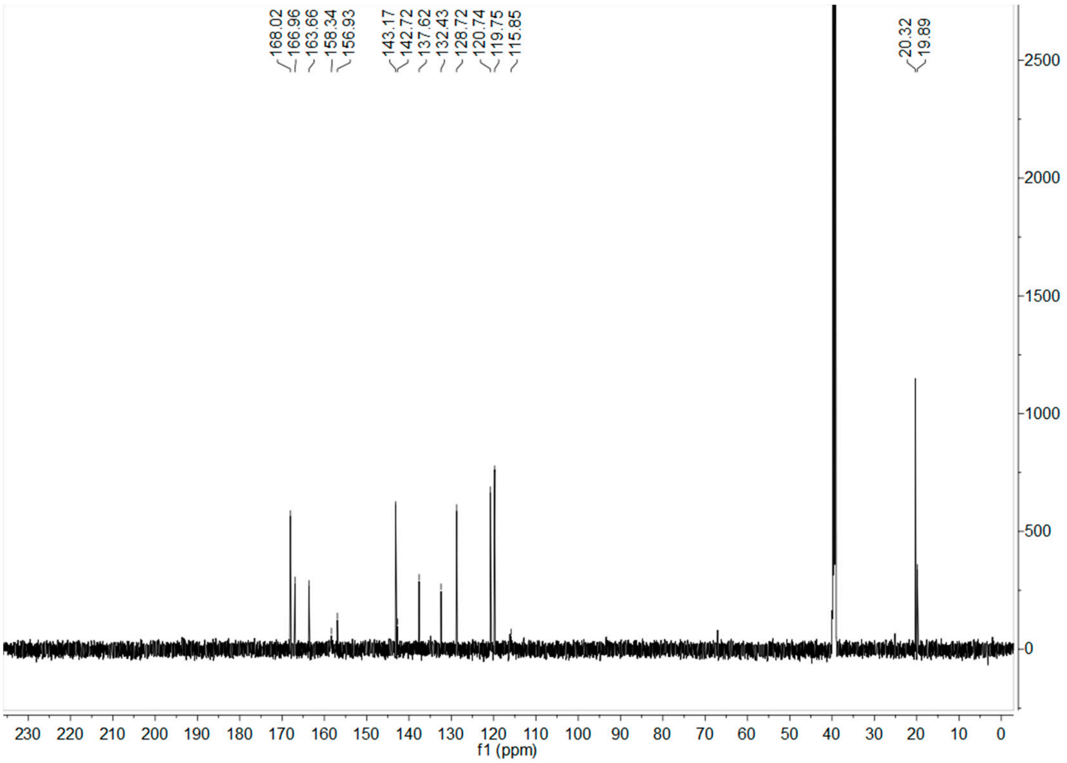

Figure S3. ^{13}C-NMR spectrum of HAMDC.
